# *MDM2 *SNP309 promoter polymorphism and *p53 *mutations in urinary bladder carcinoma stage T1

**DOI:** 10.1186/1471-2490-13-5

**Published:** 2013-01-28

**Authors:** Hans Olsson, Per Hultman, Johan Rosell, Peter Söderkvist, Staffan Jahnson

**Affiliations:** 1 Molecular and Immunological Pathology, Department of Clinical and Experimental Medicine, Faculty of Health Sciences, Linköping University, Department of Clinical Pathology and Clinical Genetics, Östergötland County Council, Linköping, Sweden; 2 Statistician, Regional Cancer Centre, Östergötland County Council, Linköping, Sweden; 3 Division of Cell Biology, Department of Clinical and Experimental Medicine, Faculty of Health Sciences, Linköping University, Linköping, Sweden; 4 Division of Urology, Department of Clinical and Experimental Medicine, Faculty of Health Sciences, Linköping University, Department of Urology, Östergötland County Council, Linköping, Sweden

## Abstract

**Background:**

Urinary bladder carcinoma stage T1 is an unpredictable disease that in some cases has a good prognosis with only local or no recurrence, but in others can appear as a more aggressive tumor with progression to more advanced stages. The aim here was to investigate stage T1 tumors regarding *MDM2* promoter SNP309 polymorphism, mutations in the *p53* gene, and expression of p53 and p16 measured by immunohistochemistry, and subsequently relate these changes to tumor recurrence and progression. We examined a cohort of patients with primary stage T1 urothelial carcinoma of the bladder and their tumors.

**Methods:**

After re-evaluation of the original slides and exclusions, the study population comprised 141 patients, all with primary stage T1 urothelial carcinoma of the bladder. The hospital records were screened for clinical parameters and information concerning presence of histologically proven recurrence and progression. The paraffin-embedded tumor material was evaluated by immunohistochemistry. Any mutations found in the *p53* gene were studied by single-strand conformation analysis and Sanger sequencing. The *MDM2* SNP309 polymorphism was investigated by pyrosequencing. Multivariate analyses concerning association with prognosis were performed, and Kaplan-Meier analysis was conducted for a combination of changes and time to progression.

**Results:**

Of the 141 patients, 82 had at least one *MDM2* SNP309 G allele, and 53 had a mutation in the *p53* gene, but neither of those anomalies was associated with a worse prognosis. A mutation in the *p53* gene was associated with immunohistochemically visualized p53 protein expression at a cut-off value of 50%. In the group with *p53* mutation Kaplan-Meier analysis showed higher rate of progression and shorter time to progression in patients with immunohistochemically abnormal p16 expression compared to them with normal p16 expression (p = 0.038).

**Conclusions:**

*MDM2* SNP309 promoter polymorphism and mutations in *p53* were not associated with worse prognosis in this cohort of patients with primary stage T1 urinary bladder carcinoma. However, patients with abnormal p16 expression and a mutated *p53* gene had a higher rate of and a shorter time to progression, and *p53* gene mutation was associated with an abnormal immunohistochemistry for p53 at a cut-off of 50%.

## Background

Urothelial carcinoma of the bladder (UCB) is an unpredictable disease, and this is particularly apparent in patients with stage T1 UCB, who are at high risk of progression (30–50%) [1,2]. The main treatment for non-muscle-invasive bladder cancer (NMIBC) is transurethral resection (TUR) combined with intravesical instillation of bacillus Calmette-Guérin (BCG). Cystectomy is the treatment of choice in patients with a higher stage of UCB (≥ T2), in spite of cystectomy, the prognosis is poor for these patients [3]. Cystectomy can be considered for NMIBC stage T1, however, performing cystectomy in every new case of stage T1 UCB is overtreatment, and hence it is an essential assignment to identify markers that can assess prognosis and aid individualization of treatment [4,5].

The molecular mechanism of tumor progression in UCB is poorly understood. Several studies have tried to explain the transformation from normal to malignant urothelium and the progression that is often seen in this disease [6]. It is known that UCB is strongly associated with alterations in the p53 pathway [7]. Mutations in the *p53* gene are often correlated with higher tumor grade and more advanced stages, as well as progression of NMIBC to muscle-invasive disease [8].

The murine double minute 2 (MDM2) is a negative regulator of p53. Furthermore, *MDM2* SNP 309 (rs2279744) promoter polymorphism has been reported to be a risk modifier in several other malignant neoplasms [9], but few studies have addressed the role of this as such a modifier in UCB, which indicates the need for further research on this subject [10].

MDM2 and p53 play a critical role in carcinogenesis [11,12]. The latter is encoded by the *p53* tumor suppressor gene, and it induces cell cycle arrest, apoptosis, DNA repair, and prevention of angiogenesis [13-15]. The *p53* gene is often mutated in malignancies, which highlights its importance in tumor development and progression. MDM2, on the other hand, is an essential negative regulator of p53, because, when present in excessive amounts, it reduces the activity of p53 via enhanced proteasomal degradation [12,16]. A single-nucleotide polymorphism (SNP309, rs2279744) located in the first intron of the core promoter region of the *MDM2* gene affects binding of the transcription factor Sp1. Sp1 binds with higher affinity to the G allele than to the T allele, which results in increased transcription of the *MDM2* gene and higher levels of MDM2 protein, and thereby inhibits the tumor suppressor function of p53 [17]. The interaction between p53 and MDM2 is a target for therapeutic intervention, and several such drugs are under development or in clinical trials [18-20].

We have previously investigated clinical and histopathological parameters, as well as immunohistochemistry (IHC) for proteins involved in cell cycle regulation ( i.e. p53, p21, pRb, p16, and cyclin D1) and for matrix metalloproteinases [21,22]. In those studies, we found that normal p53 (cut-off < 10%) was significantly associated with recurrence, and abnormal p16 was associated with progression. It is plausible that the mechanism for the latter relationship is dependent on the step involving phosphorylation of Rb. The importance of these proteins in tumor development has been described by other researchers [23-25].

In the current study, we examined a population-based cohort of patients with primary stage T1 UCB. We analyzed possible effects of the functional polymorphism SNP309 in the *MDM2* gene, and investigated the relationship between that polymorphism and mutations in the *p53* gene. We also evaluated the expression of p53, p16, pRb and p21 measured by IHC. All findings were evaluated regarding associations with the risk of progression and recurrence.

## Patients and methods

### The study population and histopathological re-evaluation

This population-based retrospective study included all patients reported in 1992–2001 to the regional Bladder Cancer Registry of the Southeast Healthcare Region in Sweden as having primary stage T1 UCB. The hospital records were reviewed to collect information concerning tumor characteristics (size and multiplicity), treatment modalities, and outcome in relation to histologically proven recurrence and/or progression, as well as eventual death from UCB. Definition of progression was recurrence with infiltration to T2 or more, regional lymph node involvement, distant metastasis, or death from bladder cancer. Re-evaluation was done by one uropathologist (HO) on the original hematoxylin-eosin-stained slides with respect to T stage [26], WHO grade [27], presence of concomitant carcinoma in situ, and lymphovascular infiltration (LVI) [28,29]. Presence of muscularis propria was required for inclusion in the study. After the re-evaluation, paraffin blocks were selected for sectioning of new tumor material for further evaluations (see below).

The study cohort initially consisted of 285 patients, and 141 remained after re-evaluation, reviewing of the hospital records, and performance of the laboratory analyses. One important reason for exclusion was a changed diagnosis in the re-evaluation (n = 52; 50 changed to Ta and two to T2). Furthermore, seven patients had missing specimens (both tissue blocks and slides), and 15 had insufficient information in their hospital records (e.g., absence of tumor characteristics and/or details of follow-up). In addition, due to a combination of poor tumor quality and methodological difficulties in processing old formalin-fixed tumor material for genetic analyses, tumor material from 70 patients was excluded from further laboratory analyses. In general, these 70 patients did not differ substantially from the 141 that remained, although, when we compared these two groups, we noted significant differences with regard to age and p21 expression measured by IHC (Table 1).

**Table 1:** **Comparison of the 60 patients that were excluded from the study with the 141 that remained **(**percentages calculated for the two groups**)

	**60 excluded**	**141 remained**	**Chi**-**square**, **significance**
Age (years)			4.08, p = 0.04
≤ 73	38%	54%	
> 73	62%	46%	
Gender			n.s.
Male	85%	82%	
Female	15%	18%	
Tumor size			n.s.
≤ 3 cm	50%	48%	
> 3 cm	50%	52%	
Tumor grade			n.s.
2	20%	14%	
3	80%	86%	
LVI*			n.s.
No	77%	68%	
Suspected	20%	23%	
Yes	3%	9%	
p21			26.6, p < 0.001
Abnormal	48%	14%	
Normal	52%	86%	
p53			n.s.
Abnormal	82%	74%	
Normal	18%	26%	
pRb			n.s.
Abnormal	88%	84%	
normal	12%	16%	
p16			n.s.
Abnormal	58%	58%	
Normal	42%	42%	
Recurrence			n.s.
No	78%	82%	
Yes	22%	18%	
Progression			n.s.
No	40%	38%	
Yes	60%	62%	

*LVI = lymphovascular invasion.

When screening the hospital records, we found that early re-resection, 4–6 weeks after initial TUR, was not stipulated as mandatory during the period that the studied material was collected, and hence this procedure had been performed on only a very small number of patients (included during the last years of the study period). The presence of a tumor at a re-resection was considered to be a recurrence. Random biopsies of normal mucosa were not performed routinely, and no patient had received intravesical BCG or chemotherapy before recurrence.

### Immunohistochemical analysis

**Figure 1:**
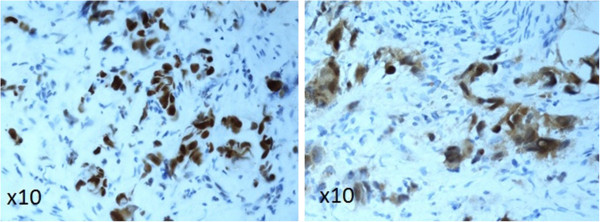
**Positive **(**abnormal**) **immunohistochemical staining for p53 **(**left**) **and p16 **(**right**) **in urinary bladder carcinoma cells.**

IHC was performed on 4-μm whole sections from the original formalin-fixed, paraffin-embedded tissue blocks. The blocks were carefully chosen, paying special attention to large tumor volume and good fixation. The sections were deparaffinized in xylene and then rehydrated, pretreated with Tris-EDTA buffer (pH 9), and stained in an automated immunostainer (DAKO TechMate-TM Horizon, DAKO Denmark A/S). For detection, we used monoclonal mouse antibodies (Table 2). Appropriate positive and negative controls were employed throughout. The antibodies were initially individually optimized with respect to the best pretreatment method and dilution. The expression levels were determined semi-quantitatively based on the fraction of tumor cells showing positive staining (0%, 1–10%, 11–25%, 26–50%, 51–75%, 76–100%). P53 was considered as abnormal when >10% positive tumor cells, p16 was considered abnormal when no (0%) or >50% positive tumor cells were found. For p53 and p16, both nuclear and cytoplasmic staining was considered as positive, although no single cases existed which just were cytoplasmic positive without being nuclei positive (see Figure 1). The cut-off values were chosen from the literature (Table 2) [30-32].

**Table 2:** Antibodies for immunohistochemistry

**Antibody**	**Clone**	**Source**	**Dilution**	**Abnormal**	**Positive**
p53	DO-7	DAKO	1:100	> 10%	Nuclei and cytoplasm
p21	SX118	DAKO	1:50	< 10%	Nuclei
p16	6H12	Novocastra	1:20	0% or > 50%	Nuclei and cytoplasm
pRb	G3-245	BD Pharmingen	1:100	0% or > 50%	Nuclei

IHC stainings for the proteins pRb and p21 were also performed using the same method as described above, and has previously been reported by our group [21].

### Genotyping by pyrosequencing for the *MDM2* gene

Two 10-μm-thick sections obtained from the original paraffin blocks were transferred to an Eppendorf tube, and DNA was purified according to the protocol provided in the Maxwell TM 16 FFPE DNA Purification Kit (Promega, Madison, WI, USA). To detect SNP309 (rs2279244), the *MDM2* gene was amplified by a PCR carried out in a reaction volume of 30 μl containing (final concentrations) 20 mM (NH4)_2_SO_4_, 75 mM Tris–HCl (pH 9.0), 0.01% Tween 20, each dNTP at 200 μM, 2.0 mM MgCl2, each primer (available on request) at 1.0 μM (Invitrogen, Paisley, UK), 0.5 U Taq DNA polymerase (Thermowhite, Saveen Werner AB, Limhamn, Sweden), and 50 ng of DNA. The amplification was performed at an annealing temperature of 60°C for 35 cycles, and the reverse primer was biotinylated. Single-stranded DNA was isolated from the PCR reaction using a Pyrosequencing Vacuum Prep Workstation (Biotage AB, Uppsala, Swden) and then transferred to a 96-well plate. The sequencing primer was annealed to the single-stranded DNA by heating the sample to 80°C for 2 min and subsequently allowing it to cool to room temperature. Thereafter, the plate was placed in the Pyrosequencing PSQ96MA system (Biotage AB, Uppsala, Sweden) for real-time sequencing and SNP detection.

Blood samples randomly collected from a population of 725 healthy individuals (registered in our database) living in the same region as the study population were tested for *MDM2* polymorphism and served as references for the frequency of such polymorphism.

### Single-strand conformation analysis and Sanger sequencing for *p53*

Tumor DNA was analyzed for mutations in exons 5–8 in the *p53* gene. PCR primers (available on request) covering the same exons and including exon/intron borders of the *p53* gene were used to generate PCR products from tumor DNA. After confirming the success of the PCR, a 1-μl aliquot of the PCR product was labeled with ^32^P-dATP in 5–10 cycles of secondary PCR using the same primers as in the primary PCR and then subjected to single-strand conformation analysis (SSCA) [33,34].

#### SSCA

Labeled PCR products were diluted 20-fold with 50% formamide/10 mM EDTA/0.1% SDS (containing xylene cyanol and bromophenol blue tracing dyes). After denaturation at 95°C for 5 min, the samples were immediately put on ice and loaded on a native 6% polyacrylamide gel containing 10% glycerol. Electrophoretic separation of single-stranded DNA was performed at 10–12 W for 16–20 h. Thereafter, the gels were attached to filter paper and dried, and then exposed to x-ray film for 16–24 h. Shifted bands (indicating a different secondary structure) were excised, and DNA was eluted and used in a re-amplification PCR reaction for direct DNA sequencing.

#### DNA sequencing

A 3–5-μl aliquot of PCR product (collected directly or from re-amplification of excised SSCA bands) was used in a standard protocol for fluorescently labeled dideoxynucleotides (BigDye, Applied Biosystems, Life Technologies), with injection into a capillary electrophoresis instrument (ABI 3500, Life Technologies) for separation and detection. The sequences that were obtained were compared with the reference sequence NC 000017 (http://www.ncbi.nlm.nih.gov), and deviations were recorded as mutations or polymorphisms. The identified mutations were confirmed by re-analysis (according to the entire procedure) from a second PCR amplification of the tumor DNA.

### Statistical analysis

Descriptive statistics were used for frequencies of mutations in *p53* and *MDM2* polymorphism. Associations between such mutations and polymorphism on the one hand and clinicopathological and immunohistochemical variables on the other were calculated by the chi-square test. Cox proportional hazards analysis was performed in a univariate and a multivariate fashion to investigate variables in relation to recurrence and progression in UCB, and to test for potential confounding variables and interactions. The results of the Cox regression are presented with hazard ratios and 95% confidence intervals. Kaplan-Meier curves were used to analyze the relative influence of IHC-determined p16 status and *p53* mutations on progression and differences were calculated with the Log rank test. All statistical analyses were performed using IBM/SPSS version 19.0. P-values of ≤ 0.05 were considered to be statistically significant, and all tests were two sided.

## Results

### Study-population

The 141 patients in the study population had a median age of 73 years (range 42–93 years) and 25 (18%) were women. Of the 141, 115 (82%) had recurrence, and 53 (38%) had progression. The intention was to follow all patients for at least 10 years. The median observation time was 65 months (4–192). The characteristics of the study-population are given in Table 3.

**Table 3:** Characteristics of the 141 patients included in the study

	***P53 *mutation**		***MDM2 *TT**	
	**No**	**Yes**	**No**	**Yes**
	88 (62%)	53 (38%)	82 (58%)	59 (42%)
Age (years)				
≤73	49 (64%)	27 (36%)	44 (58%)	32 (42%)
>73	39 (60%)	26 (40%)	38 (58%)	27 (42%)
Gender				
Male	72 (62%)	44 (38%)	66 (57%)	50 (43%)
Female	16 (64%)	9 (36%)	16 (64%)	9 (36%)
*MDM2*				
TT	38 (64%)	21 (36%)	-	-
GG	11 (61%)	7 (39%)	-	-
TG	39 (61%)	25 (39%)	-	-
*p53* mutation				
Yes	-	-	32 (60%)	21 (40%)
No	-	-	50 (57%)	38 (43%)
Recurrence				
Yes	75 (65%)	40 (35%)	66 (57%)	49 (43%)
No	13 (50%)	13 (50%)	16 (62%)	10 (38%)
Progression				
Yes	30 (57%)	23 (43%)	27 (51%)	26 (49%)
No	58 (66%)	30 (34%)	55 (62%)	33 (38%)

### *MDM2* polymorphism, *p53* mutations and IHC analysis

In all, 53 (38%) of the 141 tumors we investigated had a mutation in the *p53* gene, but there was no apparent prevalence in any particular exon, as outlined in Table 4.

**Table 4:** **Distribution of the *p53 *mutations**

**Exon**	**Number of patients**
5	13 (9.2 %)
6	9 (6.4 %)
7	13 (9.2 %)
8	18 (12.8%)
***Total***	***53(37.6%)***

The frequencies of the *MDM2* SNP309 genotypes among the 141 patients were as follows: 59 T/T (42%), 64 T/G (45%), and 18 G/G (13%). The corresponding rates in our database of 725 healthy individuals tested for *MDM2* polymorphism are 41%, 45%, and 14%, respectively. Thus the proportion of G/G individuals is nearly the same in our bladder cancer cohort and the healthy subjects. Furthermore, the genotypes are in Hardy-Weinberg equilibrium, and there is no unfavorable factor in *MDM2* polymorphism that promotes development of UCB stage T1.

We observed a significant association between *p53* mutations and p53 IHC at a staining cut-off of 50% (Chi square 8.67, p = 0.003), whereas no such relationship was seen at cut-off values lower than 50% (10%, 25%, or < 50%). There was also an association between abnormal p16 expression and *p53* mutations (Chi square 4.74, p = 0.029), and between WHO tumor grade 2 (but not to grade 3) and *p53* mutations (Chi square 6.12, p = 0.013) (data not shown). However, it should be noted that there were only 21 grade 2 tumors in the material.

No association was found when we analyzed *MDM2* polymorphism and *p53* mutations in relation to prognosis. Table 5 summarizes the results concerning progression in relation to IHC, *MDM2* polymorphism and *p53* mutations (data for recurrence not shown).

**Table 5:** **Cox proportional hazards univariate and multivariate analysis of progression after primary transurethral resection for T1 bladder carcinoma in the Southeast care region in Sweden 1992**–**2001**

	**Univariate Hazard ratio (95% CI)**	**Multivariate Hazard ratio (95% CI)**	**p-value**
LVI*			
No	1.0	1.0	
Suspected	1.01 (0.51–2.00)	0.84 (0.42–1.68)	0.70
Yes	3.10 (1.48–6.49)	3.84 (1.75–8.42)	***0***.***001***
p16			
Abnormal	1.0	1.0	
Normal	0.40 (0.23–0.74)	0.37 (0.19–0.69)	***0***.***002***
p21			
Abnormal	1.0	1.0	
Normal	1.93 (0.77–4.86)	1.39 (0.54–3.59)	0.50
p53			
Abnormal	1.0	1.0	
Normal	0.75 (0.42–1.35)	1.54 (0.84–2.82)	0.17
*p53* mutation			
No	1.0	1.0	
Yes	1.51 (0.87–2.59)	1.28 (0.73–2.23)	0.38
*MDM2* T/T			
No	1.0	1.0	
Yes	1.52 (0.89–2.62)	1.75 (1.00–3.06)	0.052
pRb			
Abnormal	1.0	1.0	
Normal	1.36 (0.70–2.65)	1.58 (0.79–3.17)	0.20

*LVI = lymphovascular invasion.

**Figure 2:**
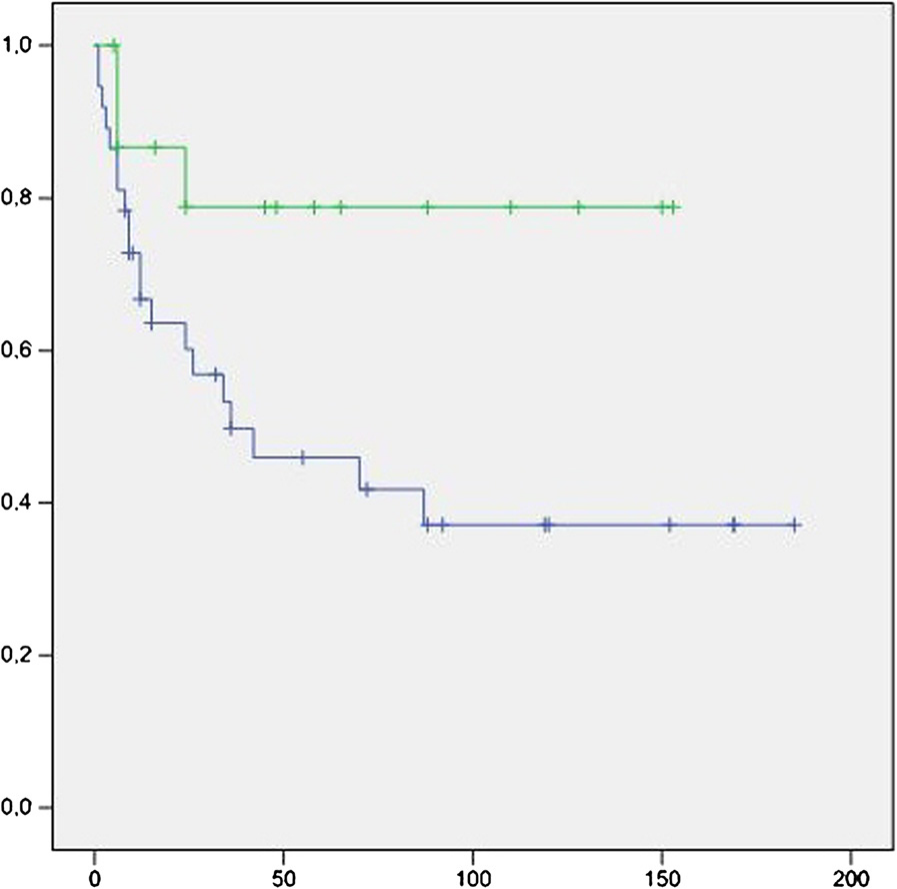
**Kaplan**-**Meier curves for patients with a *p53 *mutation showing that the time (months) to progression was significantly shorter for those with abnormal (blue curve) as compared to normal (green curve) p16 expression. **(Log rank chi square 4.32, p = 0.038).

**Figure 3:**
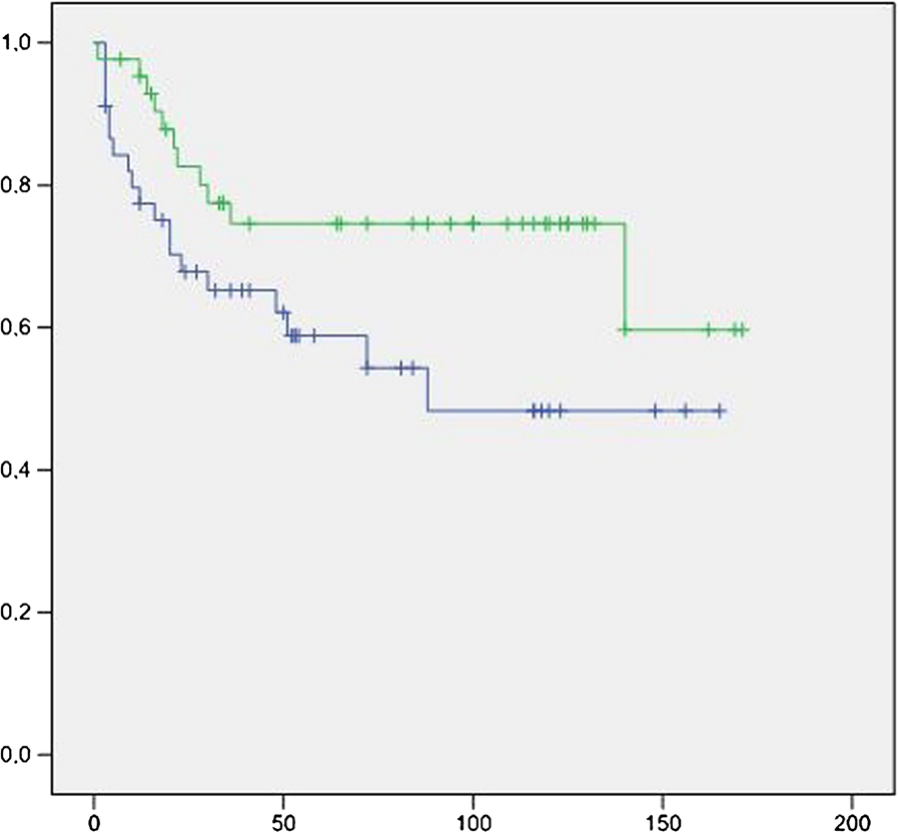
**Kaplan**-**Meier curves for patients without a *p53 *mutation showing that time (months) to progression was shorter for those with abnormal (blue curve) p16 expression as compared to those with normal (green curve) p16 expression**, **although this difference was not statistically significant. **(Log rank chi square 3.78, p = 0.052).

In patients with a *p53* mutation, Kaplan-Meier curves showed a higher progression rate and a shorter time to progression for those with abnormal p16 expression as compared to those with normal p16 expression (Log rank chi square 4.32, p = 0.038; Figure 2). For patients who lacked a *p53* mutation, no significant difference was found between those with normal and abnormal p16 expression (Log rank chi square 3.78, p = 0.052; Figure 3). However, in the whole group of UCB patients, there was an association between IHC-abnormal p16 alone and UCB progression, as previously reported by our group [21].

No association was found for pRb or p21 IHC on one side, and *MDM2* polymorphism or *p53* mutations on the other.

## Discussion

SNP309 is a common polymorphism in the regulatory region in intron 1 of *MDM2*, and it might influence the cellular levels of the p53 protein and thereby promote tumor development and progression [17]. This has been observed in several studies concerning poor outcome of various malignancies, such as lung cancer, breast cancer, gastric carcinoma, and renal cell carcinoma [35-38]. The role of a single nucleotide polymorphism in *MDM2* (i.e., SNP309) has also been evaluated in UCB, but the results have varied [10,39].

Here, we studied a large well-characterized group of patients with primary stage T1 UCB, none of whom had received intravesical BCG or chemotherapy before recurrence, a treatment strategy that is mandatory today. Accordingly, these patients exhibited a natural course of stage T1 UCB and were therefore ideal to investigate in the present context. We found that neither *MDM2* polymorphism nor *p53* gene mutations were associated with tumor progression in this study population. However, there was an association between strong IHC-determined p53 expression and *p53* gene mutations. It is debatable whether *p53* mutations can be compared with p53 expression measured by IHC. Yemelyanova et al. [40] observed concordance between *p53* gene mutations and IHC-detected p53 expression in ovarian carcinoma patients, and those investigators obtained statistically significant results at a cut-off value of 60% positive tumor cells. That value is higher than the cut-off that is usually reported as abnormal for p53 expression, which is most often 10% positive tumor cells [41]. In our investigation, a cut-off value of 50% was found to be associated with mutations, and it may reflect the mutational status of *p53* more accurately than the conventional cut-off value used for the p53 protein.

For analysis of *p53* mutations, Sanger sequencing with fluorescent dideoxynucleotides is usually not sufficiently sensitive in detecting small fractions of mutated cells, and therefore we also performed SSCA. The results showed nearly 100% agreement between SSCA mobility shifts and Sanger sequencing of excised and re-amplified SSCA bands.

We found an association between abnormal p16 expression and mutations in *p53*, which may be evidence of linkage between the p53, p16, and pRb proteins. Furthermore, this observation is supported by the results of Kaplan-Meier analysis indicating that the rate of progression and time to progression were significantly shorter in patients with IHC-abnormal p16 and *p53* mutations. Moreover, we observed that abnormal expression of p16 alone seems to be associated, with a worse prognosis, which has also been reported previously by us and by other authors [21,32,42,43].

*MDM2* polymorphism in the current UCB patients was tested in DNA from tumor tissue, and hence there was a risk of bias due to chromosomal deletions on chromosome 12 (where *MDM2* is located). However, chromosomal 12 deletions are not common in this disease, and all of the tissue specimens analyzed in our study contained normal cells, which ensured correct genotyping. Furthermore, the frequency of *MDM2* polymorphism in the UCB patients, did not differ from that noted in our reference population, and it was in Hardy-Weinberg equilibrium, which suggests that such polymorphism is stable during tumor development, and consequently there is no difference between benign and malignant cells.

Alhopuro et al. [44] studied patients with leiomyosarcoma, colorectal cancer, and squamous cell carcinoma of the head and neck, and their results indicated essentially the same proportion of individuals with *MDM2* polymorphism as noted in our investigation. Alhopuru and coworkers also reported that a healthy reference population and tumor material from their study population had approximately the same frequencies of *MDM2* polymorphism. Onat et al. [45] analyzed *MDM2* polymorphism in bladder cancer and found an association between development of the disease and *MDM2* polymorphism in patients with the G/G genotype. The frequency of *MDM2* G/G in their study was somewhat greater in the tumor population than in the control group consisting of healthy individuals, although the values those researchers obtained differ very little from the levels noted in our investigation. In an assessment of B-CLL patients, Willander et al. [46] found that overall survival was poorer in the subjects with one or two G alleles compared to those with T/T. However, the overall frequencies of G/G, T/G, and T/T found by those investigators are equivalent to the corresponding figures recorded in our study, which underlines that the *MDM2* gene is probably rather stable throughout tumor development and that one or two G alleles more likely only play a role as an unfavorable genotype.

Several studies have examined *p53* and *MDM2* polymorphism in UCB. In one of those investigations, Lu et al. [47] found that 61 (44%) of 140 patients with the disease were wild type for *p53*, whereas the corresponding proportion in our cohort was 62%, and this disparity may reflect difference in T stages between the two studies. A plausible explanation for this is that the tumors investigated by Lu and colleagues were from cystectomy specimens and thus represented higher tumor stages with more *p53* mutations (less wild-type p53). Notably, we focused on exons 5–8 in the *p53* gene, whereas Lu et al. investigated exons 2–11, although only a small number of the mutations in their study were found outside exons 5–8. Furthermore, Hernandez et al. [48] found a frequency of 65.5% *p53* mutations in a group of patients with stage T1 grade 3 UCB, but those mutations were not associated with prognosis; interestingly, exons 4–9 were evaluated in that investigation, but the majority of the mutations were found in exons 5–8.

The frequency of *p53* gene mutations varies considerably in the literature, which may reflect the general heterogeneity of stage T1 UCB as a type of malignancy. It is possible that mutations in this gene play a role in the development and progression of T1 UCB, but the definitive pathways have not yet been completely elucidated.

## Conclusions

Polymorphism in *MDM2* (SNP309) and mutations in *p53* were not associated with worse prognosis in this population-based cohort of patients with stage T1 UCB. Mutations in *p53* were associated with an IHC cut-off value of 50% for the p53 protein. Patients with IHC-abnormal p16 and *p53* mutations had a higher rate of progression and a shorter time to progression.

## Competing interests

The authors declare that they have no competing interests.

## Authors’ contributions

HO and SJ planned the study and were responsible for the design, coordination, and drafting the manuscript. PH and PS participated in the study design and helped draft the manuscript. JR and SJ performed the statistical analysis. PS and HO were responsible for the methodological issues involved in this study. All authors read and approved the final manuscript.

## Pre-publication history

The pre-publication history for this paper can be accessed here:

http://www.biomedcentral.com/1471-2490/13/5/prepub

## References

[B1] Andius P Holmang S Bacillus Calmette-Guerin therapy in stage Ta/T1 bladder cancer: prognostic factors for time to recurrence and progression BJU Int 2004 93 7 980 984 10.1111/j.1464-410X.2003.04764.x 15142147

[B2] Shahin O A retrospective analysis of 153 patients treated with or without intravesical bacillus Calmette-Guerin for primary stage T1 grade 3 bladder cancer: recurrence, progression and survival J Urol 2003 169 1 96 100 discussion 100 10.1016/S0022-5347(05)64044-X 12478112

[B3] Stein JP Radical cystectomy in the treatment of invasive bladder cancer: long-term results in 1,054 patients J Clin Oncol 2001 19 3 666 675 11157016 10.1200/JCO.2001.19.3.666

[B4] Bostrom PJ Optimal timing of radical cystectomy in T1 high-grade bladder cancer Expert Rev Anticancer Ther 2010 10 12 1891 1902 10.1586/era.10.183 21110756

[B5] Herr HW Donat SM Dalbagni G Can restaging transurethral resection of T1 bladder cancer select patients for immediate cystectomy? J Urol 2007 177 1 75 79 discussion 79 10.1016/j.juro.2006.08.070 17162005

[B6] Sjodahl G A systematic study of gene mutations in urothelial carcinoma; inactivating mutations in TSC2 and PIK3R1 PLoS One 2011 6 4 10.1371/journal.pone.0018583 PMC3077383 21533174

[B7] Smith ND The p53 tumor suppressor gene and nuclear protein: basic science review and relevance in the management of bladder cancer J Urol 2003 169 4 1219 1228 10.1097/01.ju.0000056085.58221.80 12629332

[B8] Pasin E Superficial bladder cancer: an update on etiology, molecular development, classification, and natural history Rev Urol 2008 10 1 31 43 18470273 PMC2312342

[B9] Bond GL Levine AJ A single nucleotide polymorphism in the p53 pathway interacts with gender, environmental stresses and tumor genetics to influence cancer in humans Oncogene 2007 26 9 1317 1323 10.1038/sj.onc.1210199 17322917

[B10] Horikawa Y Clinical implications of the MDM2 SNP309 and p53 Arg72Pro polymorphisms in transitional cell carcinoma of the bladder Oncol Rep 2008 20 1 49 55 18575717

[B11] Soussi T Wiman KG Shaping genetic alterations in human cancer: the p53 mutation paradigm Cancer Cell 2007 12 4 303 312 10.1016/j.ccr.2007.10.001 17936556

[B12] Knappskog S Lonning PE Effects of the MDM2 promoter SNP285 and SNP309 on Sp1 transcription factor binding and cancer risk Transcription 2011 2 5 207 210 10.4161/trns.2.5.16813 22231115 PMC3265776

[B13] Suzuki K Matsubara H Recent advances in p53 research and cancer treatment J Biomed Biotechnol 2011 2011 10.1155/2011/978312 PMC3134396 21765642

[B14] Miliani de Marval PL Zhang Y The RP-Mdm2-p53 pathway and tumorigenesis Oncotarget 2011 2 3 234 238 21406728 10.18632/oncotarget.228 PMC3260806

[B15] Ozaki T Nakagawara A p53: the attractive tumor suppressor in the cancer research field J Biomed Biotechnol 2011 2011 10.1155/2011/603925 PMC3004423 21188172

[B16] Moll UM Petrenko O The MDM2-p53 interaction Mol Cancer Res 2003 1 14 1001 1008 14707283

[B17] Bond GL A single nucleotide polymorphism in the MDM2 promoter attenuates the p53 tumor suppressor pathway and accelerates tumor formation in humans Cell 2004 119 5 591 602 10.1016/j.cell.2004.11.022 15550242

[B18] Brown CJ Reactivation of p53: from peptides to small molecules Trends Pharmacol Sci 2011 32 1 53 62 10.1016/j.tips.2010.11.004 21145600

[B19] Weber L Patented inhibitors of p53-Mdm2 interaction (2006–2008) Expert Opin Ther Pat 2010 20 2 179 191 10.1517/13543770903514129 20100001

[B20] Lauria A Molecular modeling approaches in the discovery of new drugs for anti-cancer therapy: the investigation of p53-MDM2 interaction and its inhibition by small molecules Curr Med Chem 2010 17 28 3142 3154 10.2174/092986710792232021 20666726

[B21] Olsson H Immunohistochemical Evaluation of Cell Cycle Regulators: Impact on Predicting Prognosis in Stage T1 Urinary Bladder Cancer ISRN Urologyin press 10.5402/2012/379081 PMC3523551 23304558

[B22] Olsson H Population-based study on prognostic factors for recurrence and progression in primary stage T1 bladder tumours 2012 Scand: J Urol 10.3109/00365599.2012.719539 22954205

[B23] Quentin T Altered mRNA expression of the Rb and p16 tumor suppressor genes and of CDK4 in transitional cell carcinomas of the urinary bladder associated with tumor progression Anticancer Res 2004 24 2B 1011 1023 15161057

[B24] Konecny GE Expression of p16 and retinoblastoma determines response to CDK4/6 inhibition in ovarian cancer Clin Cancer Res 2011 17 6 1591 1602 10.1158/1078-0432.CCR-10-2307 21278246 PMC4598646

[B25] Moolmuang B Tainsky MA CREG1 enhances p16(INK4a) -induced cellular senescence Cell Cycle 2011 10 3 518 530 10.4161/cc.10.3.14756 21263217 PMC3230516

[B26] Mostofi FK Davis CJ Sesterhenn IA Histological typing of urinary bladder tumours 1999 2 Berlin, Heidelberg, New York: Springer

[B27] Sobin LH, Gospodarowicz MK, Wittekind C TNM Classification of Malignant Tumours 2009 7 Oxford: Wiley-Blackwell

[B28] Cho KS Lymphovascular invasion in transurethral resection specimens as predictor of progression and metastasis in patients with newly diagnosed T1 bladder urothelial cancer J Urol 2009 182 6 2625 2630 10.1016/j.juro.2009.08.083 19836779

[B29] Algaba F Lymphovascular invasion as a prognostic tool for advanced bladder cancer Curr Opin Urol 2006 16 5 367 371 10.1097/01.mou.0000240311.08701.55 16905984

[B30] Shariat SF p53 predictive value for pT1-2 N0 disease at radical cystectomy J Urol 2009 182 3 907 913 10.1016/j.juro.2009.05.024 19616250

[B31] Hitchings AW Prediction of progression in pTa and pT1 bladder carcinomas with p53, p16 and pRb Br J Cancer 2004 91 3 552 557 10.1038/sj.bjc.6601954 15226775 PMC2409830

[B32] Shariat SF p53, p21, pRB, and p16 expression predict clinical outcome in cystectomy with bladder cancer J Clin Oncol 2004 22 6 1014 1024 10.1200/JCO.2004.03.118 14981102

[B33] Perry DJ Screening for Mutations in DNA by Single-Stranded Conformation Polymorphism (SSCP) Analysis Methods Mol Med 1999 31 105 110 21340986 10.1385/1-59259-248-1:105

[B34] Berggren P p53 mutations in urinary bladder cancer Br J Cancer 2001 84 11 1505 1511 10.1054/bjoc.2001.1823 11384101 PMC2363660

[B35] Lind H Association of a functional polymorphism in the promoter of the MDM2 gene with risk of nonsmall cell lung cancer Int J Cancer 2006 119 3 718 721 10.1002/ijc.21872 16496380

[B36] Boersma BJ Association of breast cancer outcome with status of p53 and MDM2 SNP309 J Natl Cancer Inst 2006 98 13 911 919 10.1093/jnci/djj245 16818855

[B37] Ohmiya N MDM2 promoter polymorphism is associated with both an increased susceptibility to gastric carcinoma and poor prognosis J Clin Oncol 2006 24 27 4434 4440 10.1200/JCO.2005.04.1459 16983111

[B38] Hirata H MDM2 SNP309 polymorphism as risk factor for susceptibility and poor prognosis in renal cell carcinoma Clin Cancer Res 2007 13 14 4123 4129 10.1158/1078-0432.CCR-07-0609 17634539

[B39] Shinohara A Association of TP53 and MDM2 polymorphisms with survival in bladder cancer patients treated with chemoradiotherapy Cancer Sci 2009 100 12 2376 2382 10.1111/j.1349-7006.2009.01331.x 19764997 PMC11159677

[B40] Yemelyanova A Immunohistochemical staining patterns of p53 can serve as a surrogate marker for TP53 mutations in ovarian carcinoma: an immunohistochemical and nucleotide sequencing analysis Mod Pathol 2011 24 9 1248 1253 10.1038/modpathol.2011.85 21552211

[B41] Cote RJ Elevated and absent pRb expression is associated with bladder cancer progression and has cooperative effects with p53 Cancer Res 1998 58 6 1090 1094 9515785

[B42] Benedict WF Level of retinoblastoma protein expression correlates with p16 (MTS-1/INK4A/CDKN2) status in bladder cancer Oncogene 1999 18 5 1197 1203 10.1038/sj.onc.1202452 10022125

[B43] Kruger S P16 immunoreactivity is an independent predictor of tumor progression in minimally invasive urothelial bladder carcinoma Eur Urol 2005 47 4 463 467 10.1016/j.eururo.2004.12.018 15774242

[B44] Alhopuro P The MDM2 promoter polymorphism SNP309T→G and the risk of uterine leiomyosarcoma, colorectal cancer, and squamous cell carcinoma of the head and neck J Med Genet 2005 42 9 694 698 10.1136/jmg.2005.031260 16141004 PMC1736129

[B45] Onat OE MDM2 T309G polymorphism is associated with bladder cancer Anticancer Res 2006 26 5A 3473 3475 17094469

[B46] Willander K MDM2 SNP309 promoter polymorphism, an independent prognostic factor in chronic lymphocytic leukemia Eur J Haematol 2010 85 3 251 256 10.1111/j.1600-0609.2010.01470.x 20491880

[B47] Lu ML Impact of alterations affecting the p53 pathway in bladder cancer on clinical outcome, assessed by conventional and array-based methods Clin Cancer Res 2002 8 1 171 179 11801555

[B48] Hernandez S FGFR3 and Tp53 mutations in T1G3 transitional bladder carcinomas: independent distribution and lack of association with prognosis Clin Cancer Res 2005 11 15 5444 5450 10.1158/1078-0432.CCR-05-0122 16061860

